# Insulin Receptor in Pancreatic Cancer—Crown Witness in Cross Examination

**DOI:** 10.3390/cancers13194988

**Published:** 2021-10-05

**Authors:** Steffen M. Heckl, Lukas Kercher, Samir Abdullazade, Carolin Schneider, Sandra Krüger, Hans-Michael Behrens, Susanne Sebens, Heiner Schäfer, Stefan Schreiber, Christoph Röcken

**Affiliations:** 1 Department of Internal Medicine I, University Hospital Schleswig-Holstein, 24105 Kiel, Germany; hschaef@1med.uni-kiel.de (H.S.); stefan.schreiber@uksh.de (S.S.); 2 Department of Pathology, Christian-Albrechts-University Kiel, 24105 Kiel, Germany; lukas_kercher@web.de (L.K.); samir.abdullazade@uksh.de (S.A.); stu128513@mail.uni-kiel.de (C.S.); sandra.krueger@uksh.de (S.K.); behrensm@path.uni-kiel.de (H.-M.B.); christoph.roecken@uksh.de (C.R.); 3 Department of Internal Medicine II, University Hospital Schleswig-Holstein, 24105 Kiel, Germany; 4 Institute for Experimental Cancer Research, Christian-Albrechts-University Kiel, 24105 Kiel, Germany; susanne.sebens@email.uni-kiel.de

**Keywords:** insulin receptor, pancreatic cancer, insulin, IGF1 receptor, prognosis

## Abstract

**Simple Summary:**

Pancreatic cancer (PDAC) is a highly malignant disease with low survival rates. Due to its close proximity to the insulin producing pancreatic islets, PDAC should be exposed to comparatively higher concentrations of the growth promoting hormone insulin. We wanted to know if PDAC might take advantage of this circumstance. Therefore we cross examined the insulin receptor’s (IR) role in PDAC and precursor lesions and put it into context with the expression of the insulin-like growth factor 1 receptor (IGF1R). Our study of 160 PDAC patient samples showed that IR overexpression is already present at the precursor level. IR overexpression in PDAC was associated with adverse clinical features. The IGF1R was found to play a different role than formerly assigned. We hypothesize that the close proximity to the pancreatic islets is exploited by PDAC up to the point of the islets’ ultimate destruction by local cancer growth.

**Abstract:**

Background: The proximity of pancreatic cancer (PDAC) to the physiological source of the growth promoting hormone insulin might be exploited by this highly malignant cancer entity. We investigated if (I) PDACs express the insulin receptor (IR) in cancer cells and cancer vasculature, (II) if IR correlates with clinicopathological patient characteristics, including survival, and hence is involved in PDAC biology, (III) if IR is already expressed in precursor lesions, if (IV) the IGF1 receptor (IGF1R) is associated with clinicopathological patient characteristics and survival and (V) is linked to IR expression. Methods: 160 PDAC samples were examined for IR and IGF1R expression by immunohistochemistry. A modified HistoScore was correlated with clinicopathological characteristics and survival. Results: IR overexpression was already observed in pancreatic intraepithelial neoplasia. Furthermore, it was more frequently observed in advanced disease and associated with distant metastasis, UICC stage, lymphatic invasion and an increased lymph node ratio, but without impacting survival in the end. IGF1R expression was not associated with clinicopathological parameters or survival, in contrast to former paradigms. Conclusions: We hypothesize that the close proximity to the pancreatic islets might be advantageous for cancer growth at first, but it experiences self-limitation due to surgical removal or local destruction following accelerated cancer growth.

## 1. Introduction

Pancreatic cancer is a grievous disease with limited therapeutic options and low survival rates [1,2]. Pancreatic ductal adenocarcinoma (PDAC) is the predominant pancreatic malignancy, which accounts for 90% of all cases [3]. PDAC originates from cells of the exocrine pancreas [4]. Nestled in the exocrine constituents of the pancreatic organ, the pancreatic islets fulfill their permanent task of controlling glucose homeostasis. The islets’ beta cells ensure that insulin is produced continuously and on demand and local insulin concentrations have been reported to be higher in the pancreatic microenvironment than in other organs [5].

If the natural supply of the nurturing hormone insulin was to be exploited by the most malignant cancer entity in close proximity, substantial associations with clinicopathological parameters and survival could be expected.

Fundamental evidence is provided by earlier findings with other cancer entities.

We found the insulin receptor (IR) to be overexpressed not only in cancer cells, but also in the cancer vasculature of colorectal [6] and gastric cancer [7] samples. IR overexpression was associated with clinicopathological parameters and survival.

For the IR, two isoforms—isoform B (IR-B) and isoform A (IR-A)—are known to exist [8,9,10]. IR-B confers insulin’s commonly known metabolic effects [11,12]. IR-A, on the contrary, mainly conveys proliferative signaling [13,14]. IR-A is predominantly expressed in embryonic tissue as well as in cancer cells [6,7,15,16,17,18,19] and vasculature [6,7,20]. Proliferative signaling is synergistically promoted, if the IR-A is co-expressed with the insulin-like growth factor 1 receptor (IGF1R) [15,21]. The IGF1R has been described to be expressed in PDAC and has been associated with worse survival [22]. The IGF1R and the IR-A may synergistically form hybrid receptors, thereby enabling the resulting IGF1R-IR-A-hybrid to be stimulated by IGF1 as well [15]. The reported negative impact of IGF1R expression on PDAC patient survival [22] and the synergism between the IGF1R and IR described for other cancer entities gives reason to suspect that the IR plays a role in PDAC biology and outcome. Until now, it is unknown whether IR expression in PDAC is associated with clinicopathological parameters or survival.

In this study, we intended to cross examine the role of the IR in PDAC and precursor lesions and put it into context with IGF1R expression. We therefore tested the following hypotheses: (I) PDACs express the IR in cancer cells (CC-IR) and cancer vasculature (VIR). (II) The expression of the IR in PDAC correlates with clinicopathological patient characteristics, including survival. (III) IR expression already occurs at the level of precursor lesions, namely pancreatic intraepithelial neoplasia (PanIN). (IV) The expression of IGF1R in PDAC is associated with clinicopathological patient characteristics and survival and (V) is linked to the expression of the IR.

## 2. Materials and Methods

### 2.1. Study Population and Histology

From the archive of the Department of Pathology, University Hospital Schleswig-Holstein, Kiel, Germany, we retrieved all patients with PDAC who had undergone a surgery (Whipple procedure) for PDAC resection or had received a diagnostic biopsy between 1999 and 2017. Before the respective procedures, all patients had given written informed consent for a possible future scientific use of their biological material. Ethical approval was obtained from the local ethical review board (D 499/18) of the University Hospital Schleswig-Holstein, Kiel, Germany, which permitted us to use the patient material. Patients were included if a PDAC was confirmed by histology. Samples were excluded if a tumor type other than PDAC was identified. Gross sectioning and histological examination were performed by trained and board certified surgical pathologists. The Epidemiological Cancer Registry of the state of Schleswig-Holstein, Germany, provided the date of patient death and the cause of death and distinguished between deaths from other causes and tumor-related deaths. After study inclusion, all patient data were pseudonymized.

### 2.2. Histology

Following fixation in neutral buffered formalin, all tissue specimens were embedded in paraffin. The specimens were sectioned, deparaffinized and subsequently stained with hematoxylin and eosin. The World Health Organization criteria were used for histological classification. The pTNM-stage of all study patients was determined according to the 8th edition of the UICC guidelines [23]. The WHO classification of tumors—digestive system tumors, 5th edition [24], served to classify PanIN into low versus high grade lesions.

### 2.3. Immunohistochemistry

Immunohistochemistry was performed with monoclonal antibodies directed against CD31 (dilution 1:100; mouse monoclonal antibody; JC70; Cell Marque, Rocklin, CA, USA) using the autostainer Bond™ Max System (Leica Microsystems GmbH, Wetzlar, Germany) according to the manufacturer’s instructions. Antigen retrieval was carried out with the ER2 buffer (EDTA-buffer Bond pH 9.0). The Bond™ Polymer Refine Detection Kit (DS 9800; brown labelling; Novocastra; Leica Microsystems GmbH, Wetzlar, Germany) was employed for the immunoreaction.

IR and IGF1R immunostaining were both carried out manually. For IR immunostaining, a rabbit monoclonal anti-insulin receptor β-antibody (dilution 1:50; clone 4B8; Cell Signaling Technologies, Danvers, MA, USA) was used, which detects both IR isoforms; for IGF1R immunostaining, a rabbit monoclonal IGF1-receptor β antibody (dilution 1:50; clone D406W; Cell Signaling Technologies, Danvers, MA, USA) was chosen. Primary antibody incubation was performed overnight at 4 °C. Identical immunostaining protocols were carried out for both immunostaining reactions:

Following deparaffinization, all sections were boiled in EDTA buffer (pH 9.0; 1 min; 125 °C), then washed with Tris-buffered saline (TBS) and then treated with hydrogen peroxide block (Thermo Fisher Scientific) for 15 min, washed with TBS and then blocked with Ultra V Block (Thermo Fisher Scientific) for 5 min. The ImmPRESS reagent peroxidase universal anti-mouse/rabbit Ig—MP-7500 and the ImmPact NovaRed peroxidase substrate SK-4805 Kit (Vector Laboratories, Burlingame, CA, USA, respectively) were used for the visualization of immunoreactions. Subsequently, counterstaining with hematoxylin was carried out. The omission of the primary antibody served as negative controls. Healthy endometrium samples (proliferative phase) were used as positive controls.

### 2.4. Evaluation of CD31-Immunostaining

The CD31-immunostaining was evaluated in order to confirm the presence of cancer vasculature, i.e., especially the presence of capillaries, within the respective samples. Cancer vasculature was defined as capillaries, venules and arterioles surrounded by PDAC cancer cells.

### 2.5. Evaluation of IR and IGF1R Immunostaining

A modified HistoScore (HScore) was used to evaluate the immunostaining of the IR and IGF1R, respectively: First, the staining intensity of the respective cells was judged. A score of 0 (no staining evident), 1+ (weak) and 2+ (strong immunostaining present) was established. Secondly, the percentage of cells with no (0), weak (1+) or strong (2+) immunostaining was evaluated. For each PDAC sample, the percentages added up to 100%. A sample with strong immunostaining (2+) in all cancer cells was categorized as 100% “2+” and a case with week immunostaining (1+) in one half and absent immunostaining (0) in the other half of the sample was classified as 50% “1+” and 50% “0”. An HScore was calculated using the formula: HScore = [0 × percentage of immunonegative tumor cells] + [1 × percentage of weakly stained tumor cells] + [2 × percentage of strongly stained tumor cells]. If all cancer cells within a tumor sample displayed strong immunostaining, the maximal HScore = 200 was achieved according to the formula: [0 × 0%] + [1 × 0%] + [2 × 100%] = 200. Weak immunostaining in all of the cancer cells would yield an HScore of [0 × 0%] + [1 × 100%] + [2 × 0%] = 100. The multipliers serve to improve the stratification of the HScores, as samples with high and those with low immunostaining intensity are more distinguishably separated.

All PDAC samples were screened and representative cases were selected to serve as reference standards for IR immunostaining intensity (IR 0, IR 1+ and IR 2+) as well as for IGF1R immunostaining intensity (IGF1R 0, IGF1R 1+ and IGF1R 2+).

IR immunostaining was evaluated for cytoplasmic (cCC-IR) and membranous (mCC-IR) expression in tumor cells and vascular (VIR) IR expression in cancer vasculature. IGF1R immunostaining was evaluated for cytoplasmic (c-IGF1R) and membranous (m-IGF1R) expression in cancer cells, but not in vessels, as the IGF1R is not expressed in endothelial cells. Finally, a median HScore was calculated for each parameter and served as a cut-off to distinguish between high versus low IR- or IGF1R expression, respectively.

PanIN lesions were regularly found within PDAC samples. Their IR and IGF1R expression profiles were separately evaluated. The evaluation of PanINs including the calculation of a respective HScore was identical to the process described above for the surrounding cancer tissue. In order to ensure comparability between cancer and PanIN sites, we defined the median HScore of the PDAC collective to be the reference for the classification of PanIN-HScores into high versus low.

### 2.6. Statistical Analyses

For statistical analyses, SPSS version 24.0 (IBM Corp., Armonk, NY, USA) was used. The correlation between non-ordinal clinicopathological patient characteristics and the VIR status, the CC-IR status or the IGF1R status was tested with Fisher’s exact test. T category, N category, UICC stage and tumor grading as variables of ordinal scale were tested with Kendall’s tau test. The Kaplan–Meier method served to determine median survival with 95% confidence intervals. The log-rank test was used to test differences between median survivals. A multivariate survival analysis (Cox regression) was conducted. A *p* value of ≤0.05 was considered to be significant. All *p* values are displayed uncorrected. We applied the Siemes (Benjamini–Hochberg) procedure to compensate false discovery rate within the correlations. *p* values having lost significance are highlighted.

## 3. Results

### 3.1. Characteristics of the Study Population

The clinicopathological patient characteristics of the PDAC cohort are summarized in Table 1 and Table 2. One hundred and sixty patients fulfilled all study criteria and were included in the study.

### 3.2. Immunohistochemical Detection of IR and IGF1R in PDAC Tissues

**Figure 1:**
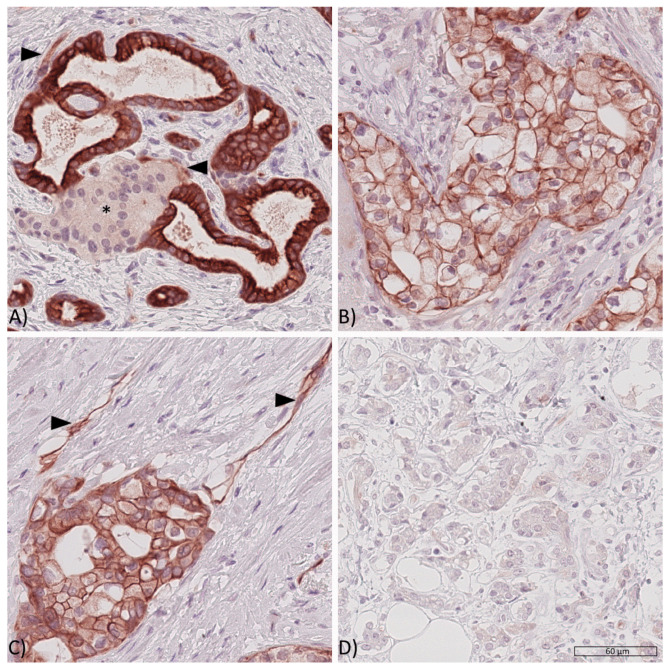
Expression of the insulin receptor in PDAC tissues. Representative PDAC tissue samples showing (**A**) high cytoplasmic (cCC-IR 2+), high membranous (mCC-IR 2+) and low vascular (arrow heads, VIR 1+) insulin receptor expression with pancreatic cancer cells surrounding a pancreatic islet (asterisk*), (**B**) low cytoplasmatic (cCC-IR 1+) and low (mCC-IR 1+) as well as high membranous (mCC-IR 2+) insulin receptor expression, (**C**) low (mCC-IR 1+) as well as high (mCC-IR 2+) membranous, weak cytoplasmic (cCC-IR 1+) and strong vascular (VIR 2+, arrowheads) insulin receptor expression and (**D**) absent tumoral or vascular insulin receptor expression. Anti-insulin receptor immunostaining, hematoxylin counterstaining. Original magnification A-D: 400×.

**Figure 2:**
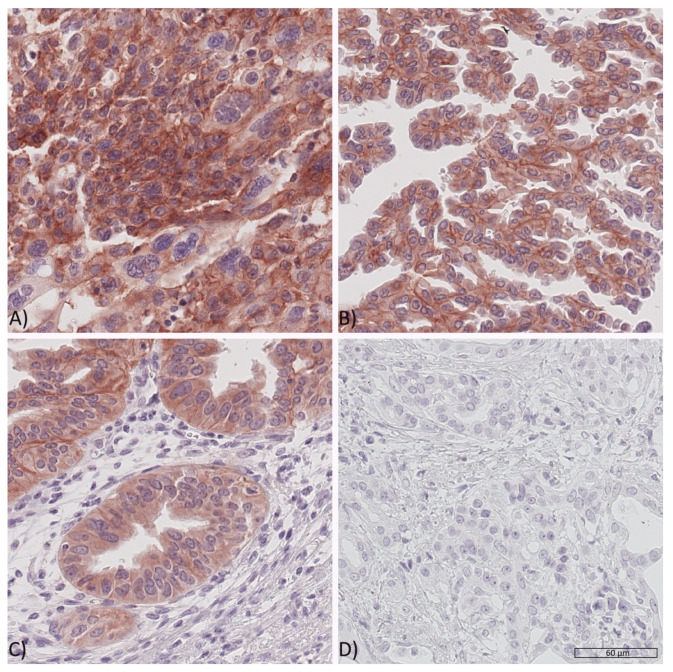
Expression of the IGF1 receptor in PDAC tissues. Representative PDAC tissue samples showing (**A**) high cytoplasmic (c-IGF1R 2+), high membranous (m-IGF1R 2+) IGF1 receptor expression in tumor cells, (**B**) weak cytoplasmic (c-IGF1R 1+) and weak as well as high membranous (mIGF1R 1+ and 2+) IGF1 receptor expression, (**C**) showing weak cytoplasmic (c-IGF1R 1+) and occasionally high membranous (upper left corner, m-IGF1R 2+) IGF1 receptor expression and (**D**) no sign of IGF1 receptor expression (IGF1R 0). Original magnification A-D: 400×.

Whole tissue sections were used to study IR and IGF1R expression. The three possible staining intensities were observed in varying extents and combinations within one respective sample (Figure 1 and Figure 2).

cCC-IR, mCC-IR, VIR, m-IGF1R and c-IGF1R were found to be heterogeneously expressed within the cancer site. Therefore, areas with strong, weak or absent immunostaining could be found side by side—with a varying percental coverage—within one respective tumor sample. The HScore acknowledged tumor heterogeneity by accounting for the percental proportion of each staining category. Dichotomization of the tumor sample HScores served to rank the tumors as low or high.

Given that percental proportions of each staining category varied within one given sample, tumor cells with a weak cytoplasmic immunostaining (cCC-IR 1+) were found within 158 (98.75%) samples and those with a strong cytoplasmic immunostaining (cCC-IR 2) were seen in 145 (90.6%) cases. Tumor cells devoid of any cytoplasmic IR immunostaining were seen in 2 (1.25%) cases. The median HScore for cCC-IR was 101 (range 0–186) and the study group was dichotomized into cCC-IR low (HScore < 101) and cCC-IR high (HScore ≥ 101). Dichotomization revealed 79 cases (49.4%) as cCC-IR low and 81 cases (50.6%) as cCC-IR high.

Given that percental proportions of each staining category varied within one given sample, tumor cells with a weak membranous immunostaining (mCC-IR 1+) were present in 157 (98.1%) and those with a strong immunostaining (mCC-IR 2+) were observed in 151 (94.4%) samples. Cancer cells lacking any immunostaining (mCC-IR 0) were seen in 149 (93.1%) cases. The median HScore for mCC-IR was 120 (range 0–192) and the collective was dichotomized into mCC-IR low (HScore < 120) and mCC-IR high (HScore ≥ 120). 75 (46.9%) PDAC samples were categorized as mCC-IR low and 85 (53.1%) as mCC-IR high.

CD31 immunostaining confirmed the presence of capillaries for further evaluation of VIR expression in all tumor sections. VIR was exclusively found within the tumor and not within the surrounding non-neoplastic tissue. VIR was predominantly seen in capillaries and only to a lesser degree in venules or arterioles. VIR showed weak immunostaining (VIR 1+) in 149 (93.1%) and strong immunostaining (VIR 2+) in 145 (90.6%) samples. Cancer vessels with absent vascular immunostaining were seen in 138 (86.3%) cases. The median HScore for VIR was 135 (0–200), which was used for dichotomization into VIR low (HScore < 135) and VIR high (HScore ≥ 135). 77 (48.1%) samples were classified as VIR low and 83 (51.9%) as VIR high.

Some tumor cells were seen to have weak cytoplasmic IGF1R immunostaining (c-IGF1R 1+) in 121 (75.6%) cases and strong immunostaining (c-IGF1R 2+) in 41 (25.6%) cases. Cancer cells without any cytoplasmic IGF1R immunostaining (c-IGF1R 0) were observed in 157 (98.1%) samples. The median HScore for c-IGF1R was 10 (0–140), which served for dichotomization into c-IGF1R low (HScore < 10) and c-IGF1R high (HScore ≥ 10). Seventy-six (47.5%) cases were grouped as c-IGF1R low and 84 (52.5%) cases as c-IGF1R high.

Given that percental proportions of each staining category varied within one given sample, cancer cells with a weak membranous IGF1R immunostaining (m-IGF1R 1+) were detected in 123 (76.9%) and cancer cells with a strong membranous immunostaining (m-IGF1R 2+) were seen in 91 (56.9%) of all samples. Cancer cells devoid of membranous IGF1R immunostaining (m-IGF1R 0) were observed in 158 (98.8%) cases. The median HScore for m-IGF1R was 12 (0–160) and was used for dichotomization into m-IGF1R low (HScore < 12) and m-IGF1R high (HScore ≥ 12). Seventy-nine (49.4%) samples were classified as m-IGF1R low and 81 (50.6%) cases were classified as m-IGF1R high.

In Contrast to the IR, no IGF1R Expression Was Detected in the Vasculature.

### 3.3. Correlation of Insulin Receptor and IGF1 Receptor Expression in Cancer Cells and Vessels in PDAC Tissues

VIR high correlated significantly with m-IGF1R high as well as c-IGF1R high (*p* = 0.017 and *p* = 0.011; Table 3). Significance was lost upon multiple testing. No correlations were found between CC-IR and IGF1R expression in cancer cells. Expression of VIR and cCC-IR (*p* = 0.429) or mCC-IR (*p* = 0.635) were also not correlated.

### 3.4. Correlation of Insulin Receptor Expression with Clinicopathological Patient Characteristics

In order to examine the potential clinical role of IR expression in PDAC we correlated cCC-IR, mCC-IR and VIR expression with clinicopathological patient characteristics (Table 1). cCC-IR-high was significantly associated with advanced UICC stages, distant metastasis and lymphatic invasion. Significance was lost upon multiple testing. While mCC-IR expression was not associated with clinicopathological patient characteristics, VIR-high expression significantly correlated with a high lymph node ratio and tended to be expressed in tumors of higher grading categories. Multiple testing led to the loss of statistical significance.

### 3.5. Correlation of IGF1 Receptor Expression with Clinicopathological Patient Characteristics

c-IGF1R-high tended to be associated with lymphatic invasion, but this was not significant. No further significant associations between either c-IGF1R or m-IGF1R and clinicopathological patient characteristics were found (Table 2).

### 3.6. Correlation of Diabetes and Insulin Receptor/IGF1 Receptor Expression in PDAC Patients

Information regarding the presence or absence of diabetes mellitus was available for 101 out of 160 PDAC patients. The retrospective analysis of available oncologic patient files showed that 22.8% suffered from type 2 diabetes and an additional 5% had developed a post-operative type 3 diabetes. However, no association was found between diabetes and IR or IGF1R expression in PDAC patients (Table 1 and Table 2).

### 3.7. Survival Analysis

**Figure 3:**
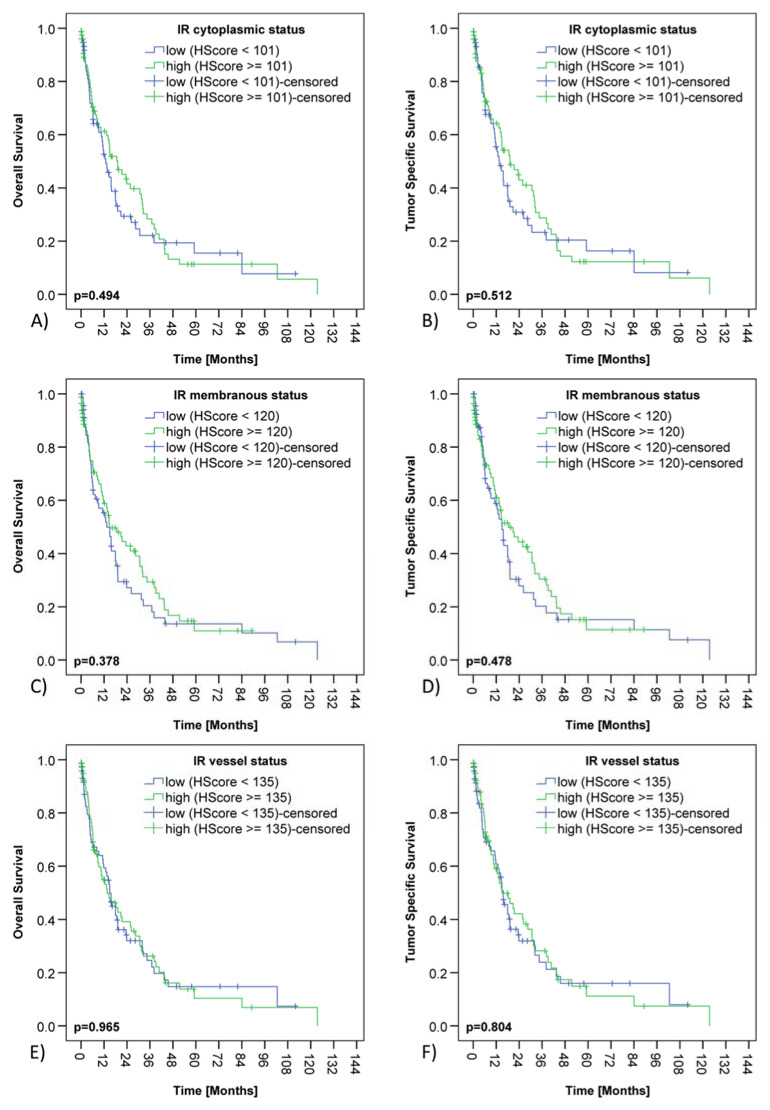
Insulin receptor expression and survival of PDAC patients. Kaplan–Meier curves demonstrating correlations between cytoplasmic insulin receptor expression in cancer cells (cCC-IR) and overall (**A**) (*p* = 0.494) as well as tumor specific survival (**B**) (*p* = 0.512). Kaplan–Meier curves showing correlations between membranous insulin receptor expression in tumor cells (mCC-IR) and overall (**C**) (*p* = 0.378) as well as tumor specific survival (**D**) (*p* = 0.478). Kaplan–Meier curves depicting correlations between insulin receptor expression in tumor vasculature (VIR) and overall (**E**) (*p* = 0.965) as well as tumor specific survival (**F**) (*p* = 0.804).

**Figure 4:**
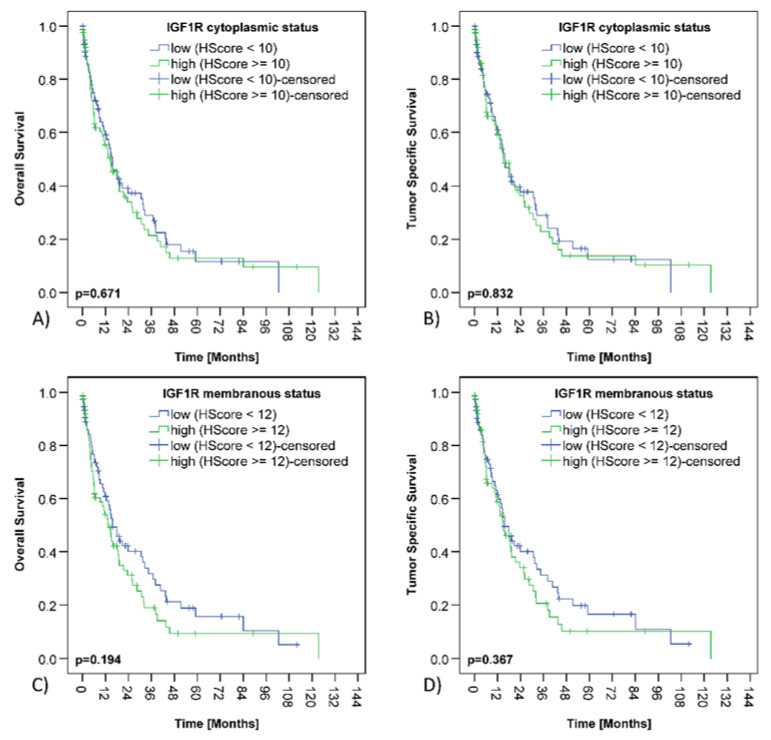
IGF1 receptor expression and survival of PDAC patients. Kaplan–Meier curves displaying correlations between cytoplasmic IGF1 receptor expression in cancer cells (c-IGF1R) and overall (**A**) (*p* = 0.671) and tumor specific survival (**B**) (*p* = 0.832). Kaplan–Meier curves presenting correlations between membranous IGF1 receptor expression in cancer cells (m-IGF1R) and overall (**C**) (*p* = 0.194) and tumor specific (**D**) (*p* = 0.367) survival.

The median overall survival (OS) of the PDAC collective was 14.9 months and the median tumor specific survival (TSS) was 15.7 months. cCC-IR high, mCC-IR high, c-IGF1R high or m-IGF1R high did not have any impact on survival (Table 1 and Table 2; Figure 3 and Figure 4).

### 3.8. Insulin and IGF1 Receptor Expression in Pancreatic Intraepithelial Neoplasia (PanIN)

**Figure 5:**
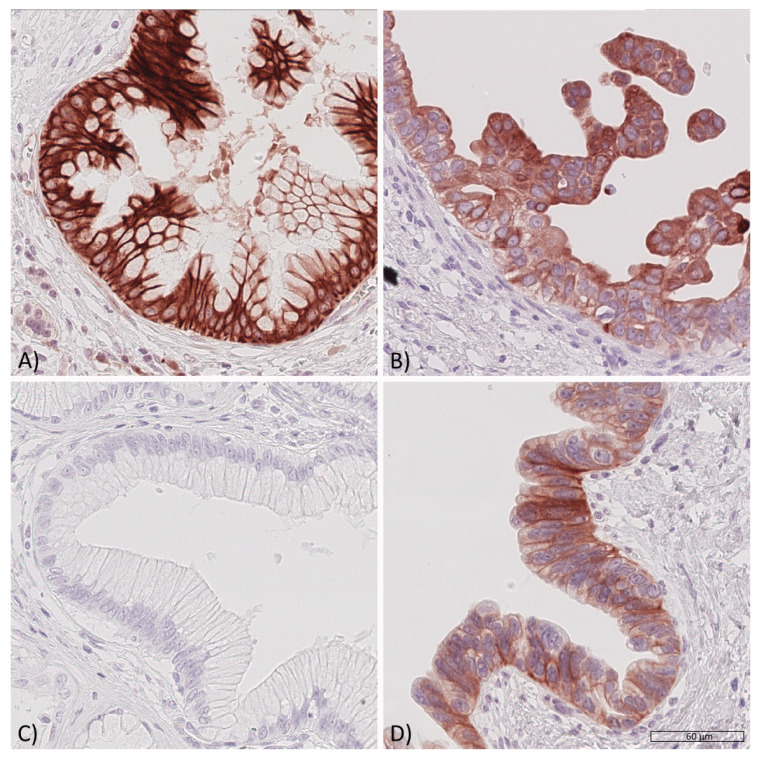
Expression of the insulin receptor and of the IGF1 receptor in pancreatic intraepithelial neoplasia (PanIN). Representative samples of pancreatic intraepithelial neoplasia (PanIN) showing insulin receptor and IGF1 receptor expression. A low-grade PanIN lesion with (**A**) high cytoplasmic (cCC-IR 2+) and high membranous (mCC-IR 2+) insulin receptor expression, a high-grade PanIN lesion (**B**) with low (cCC-IR 1+) as well as partially high cytoplasmic (cCC-IR 2+) insulin receptor expression and focally high membranous (mCC-IR 2+) insulin receptor expression, a low-grade PanIN lesion (**C**) lacking IGF1 receptor expression and a high-grade PanIN lesion (**D**) showing low cytoplasmic (c-IGF1R 1+) and high membranous (m-IGF1R 2+) IGF1 receptor expression. Original magnification A-D: 400×.

Within all PDAC samples, 40 lesions were identified as low grade PanIN and 14 lesions were classified as high grade PanIN. cCC-IR high, mCC-IR high, m-IGF1R high as well as c-IGF1R high were observed in PanIN lesions (Figure 5, Table 4). m-IGF1R expression in high grade PanIN lesions correlated with m-IGF1R expression of the surrounding tumor tissue (*p* = 0.031; Table 4; loss of significance upon multiple testing). No further correlations were found between PDAC samples and intratumoral PanIN lesions with regard to the IR/IGF1R expression profile (Table 4).

## 4. Discussion

The cross examination of the IR’s expression profile in PDAC lead to intriguing results and shed a new light on the IR-/IGF1-R-axis in cancer. We speculated that the high insulin levels present within the pancreatic organ especially predestine PDACs and their precursor lesions to IR overexpression. From an evolutionary perspective, it seems conclusive that particularly IR-overexpressing PDACs benefit from insulin production at close proximity to pancreatic islet cells, enabling continuous stimulation.

Type 2 diabetes has been described to be a risk factor for PDAC development throughout the literature [25,26]. Recently, Rahn et al. [27] demonstrated that hyperglycemia promotes the acquisition of malignancy associated alterations in PDAC in vitro and in vivo. Although diabetes-mediated hyperinsulinemia is a clear suspect of pancreatic carcinogenesis as well [26], the role of the IR is still insufficiently understood. The fact that our study did not identify any associations between IR expression and diabetes in PDAC patients might be explained by the dataset of oncologic patient files, which were not designed to answer this particular question and have therefore been incomplete in this regard.

Until now, the role of the IGF1R in PDAC seemed to be clear. With regard to many cancer entities—including colorectal or pancreatic cancer—the IGF1R was assigned the role of the villain. We already demonstrated for colorectal cancer that this role had been wrongfully assigned [28] and that this might explain why trials with IGF1R inhibitors had failed in this cancer entity. The same seems to be true for PDAC: Although former studies demonstrated decreased survival for PDAC patients with elevated IGF1R expression [22], IGF1R inhibitors did not improve prognosis of patients with this cancer entity [29]. In our study, IGF1R expression was not associated with diminished survival, therefore contrasting the results of another study group [22]. The reasons for the discrepancy might root in different patient cohorts or different evaluation systems: The group of Hirakawa et al. [22] used a scoring system ranging from 0 (no immunoreaction or immunoreaction in <10% of tumor cells) to 3 (strong immunoreaction in >10% of tumor cells); scores of 2+ and 3+ were considered to be positive for IGF1R overexpression. In our scoring system, the percentage of IGF1R positive tumor cells was quantified in a more concise manner and we only distinguished between immunostaining intensity scores ranging from 0 to 2 in order to avoid a potential error of central tendency. Additionally, the calculation of the HScore might also make a difference; however, the scoring system has proven itself in previous studies [7,28]. In detail, the HScore serves to consider tumor heterogeneity and to improve dichotomization into low and high receptor expression.

IR overexpression was observed in precursor lesions and was predominantly seen in patients with advanced disease at the time of diagnosis. We hypothesize that high local insulin concentrations present within the pancreatic organ stimulate the growth of precursor lesions and of PDAC via direct as well as indirect mechanisms. Besides direct stimulation of PDAC growth through the mitogenic IR-A, other, proliferation independent, mechanisms are involved: We recently found that the IR and the PD-L1 receptor are overexpressed in PDAC samples and demonstrated insulin-mediated PD-L1 inducibility with consecutive T-cell-suppression in co-culture experiments [30]. This mechanism was shown in a small fraction of PDAC patients. Out of these, PD-L1 and IR co-expressing patients had shown a T3 stage and nodal spread at the time of diagnosis and some of them had already metastasized. IR/PD-L1 coexpression might facilitate cancer progression by favoring immune evasion in a subset of PDAC patients and needs to be further examined in future studies.

The involvement of the tumor microenvironment (TME) is further underscored by the observations made by Ireland et al. [31] who associated the infiltration of tumor-associated macrophages (TAM) with the IR/IGF1-R-axis in a small PDAC collective. Ireland et al. stained PDAC samples for activated IR/IGF1R by using an antibody that binds both target receptors in a phosphorylated state. CD68+/CD163+ TAMs were found to surround IR/IGF1R-stained PDAC tumor cells. The results were reproduced by the group in a murine PDAC orthotopic model. TAMs and myofibroblasts were identified to be major producers of IGF1 and IGF2. Both are ligands of the IGF1R, but also of the IR-A. IGF inhibition improved the response to gemcitabine in a preclinical PDAC mouse model, but IGF inhibition alone only modestly affected PDAC tumor growth. A combination of 5-FU or paclitaxel with the IGF inhibitor only yielded a minor decrease in tumor growth. No clinical or patient survival data had been provided by the group. Potential interactions between the IR and TME are mostly uncharted territory and demand future studies.

The association between IR expression and a progressed disease at the time of diagnosis might additionally root in interactions between the IR and other tyrosine kinase receptors—such as observed in gastric cancer with the HER2 receptor [7]—and has to be closely looked at.

We have demonstrated for the first time that IR expression is associated with clinicopathological parameters in PDAC, but surprisingly, IR expression was not associated with survival in PDAC patients. These findings contrast the observations made in gastric cancer [7] or colorectal cancer [6], in which the IR was significantly associated with survival. We suspect the underlying mechanism to be linked to PDAC’s unique local origin.

IR overexpression might promote PDAC growth as outlined above, but accelerated local growth also implies an accelerated destruction of the pancreatic islets which are the source of the hormone insulin. Both local destruction as well as an instantaneous surgery if still possible at the time of diagnosis lead to the removal of the possibly crucial proximity between pancreatic islets and IR-overexpressing PDAC cells. The future fate of PDAC patients usually involves metastasis, but IR-overexpressing metastases might not have the same necessary degree of stimulation any more due to comparatively diminished local insulin concentrations. This might represent the turning point in the natural course of IR-expressing PDAC and might explain the allegedly opposing observation of adverse clinicopathological parameters and an ultimately unchanged survival in the end. Future cross examination will be necessary.

## 5. Conclusions

IR overexpression in cancer cells and vasculature of PDAC patients is more frequently found in advanced disease. Potential entanglements of the IR with the TME and other tyrosine kinase receptors are to be expected and to be examined in the future. We hypothesize that the contribution of the IR/IGF1R-axis to PDAC cancer growth experiences a self-limitation either by the local destruction of pancreatic islets via local destructive growth or by the surgical removal of the primary cancer. The close proximity to pancreatic islets as insulin’s natural source might represent an advantage for IR-overexpressing PDAC at first, but the loss or removal thereof might prevent a diminished survival in the end. Future trials will be necessary.

## Figures and Tables

**Table 1:** Correlation between clinicopathological patient characteristics and the expression of the insulin receptor (IR) in cancer vessels and cells in PDAC tissues.

		Total		IR Vascular Expression				Cytoplasmic IR Expression				Membranous IR Expression			
				Low HScore < 135		High HScore ≥ 135		Low Hscore < 101		High HScore ≥ 101		Low HScore < 120		High HScore ≥ 120	
		*n*	(%)	*n*	(%)	*n*	(%)	*n*	(%)	*n*	(%)	*n*	(%)	*n*	(%)
**Gender**	*n p*-Value _(a)_	160		160			1.000	160			0.527	160			0.057
Male		80	(50.0)	39	(48.8)	41	(51.2)	37	(46.3)	43	(53.8)	31	(38.8)	49	(61.3)
Female		80	(50.0)	38	(47.5)	42	(52.5)	42	(52.5)	38	(47.5)	44	(55.0)	36	(45.0)
**Age Group**	*n p*-Value _(a)_	160		160			1.000	160			0.206	160			0.342
<68.3 years		80	(50.0)	39	(48.8)	41	(51.2)	35	(43.8)	45	(56.3)	41	(51.2)	39	(48.8)
≥68.3 years		80	(50.0)	38	(47.5)	42	(52.5)	44	(55.0)	36	(45.0)	34	(42.5)	46	(57.5)
**T-Category**	*n p*-Value _(b)_	147		147			0.729	147			0.315	147			0.278
T1		4	(2.7)	2	(50.0)	2	(50.0)	2	(50.0)	2	(50.0)	1	(25.0)	3	(75.0)
T2		9	(6.1)	5	(55.6)	4	(44.4)	6	(66.7)	3	(33.3)	4	(44.4)	5	(55.6)
T3		130	(88.4)	61	(46.9)	69	(53.1)	69	(53.1)	61	(46.9)	63	(48.5)	67	(51.5)
T4a/b		4	(2.7)	2	(50.0)	2	(50.0)	1	(25.0)	3	(75.0)	3	(75.0)	1	(25.0)
**lymph node ratio**	*n p*-Value _(a)_	147		147			0.048 *	147			0.869	147			0.742
low (<0.133)		73	(49.7)	41	(56.2)	32	(43.8)	38	(52.1)	35	(47.9)	34	(46.6)	39	(53.4)
high (≥0.133)		74	(50.3)	29	(39.2)	45	(60.8)	40	(54.1)	34	(45.9)	37	(50.0)	37	(50.0)
***N*-Category**	*n p*-Value _(a)_	147		147			0.674	147			0.527	147			1.000
N0		27	(18.4)	14	(51.9)	13	(48.1)	16	(59.3)	11	(40.7)	13	(48.1)	14	(51.9)
*n*+ (N1, N2)		120	(81.6)	56	(46.7)	64	(53.3)	62	(51.7)	58	(48.3)	58	(48.3)	62	(51.7)
**M-Category**	*n p*-Value _(a)_	135		135			1.000	135			0.026 *	135			0.289
M0		120	(88.9)	53	(44.1)	67	(55.8)	63	(52.5)	57	(47.5)	54	(45.0)	66	(55.0)
M1		15	(11.1)	7	(46.7)	8	(53.3)	3	(20.0)	12	(80.0)	9	(60.0)	6	(40.0)
**UICC Stage**	*n p*-Value _(b)_	129		129			0.971	129			0.032 *	129			0.434
IA		3	(2.3)	2	(66.7)	1	(33.3)	2	(66.7)	1	(33.3)	1	(33.3)	2	(66.7)
IB		2	(1.6)	1	(50.0)	1	(50.0)	1	(50.0)	1	(50.0)	2	(100.0)	0	(0.0)
IIA		14	(10.9)	6	(42.9)	8	(57.1)	8	(57.1)	6	(42.9)	5	(35.7)	9	(64.3)
IIB		92	(71.3)	39	(42.4)	53	(57.6)	51	(55.4)	41	(44.6)	43	(46.7)	49	(53.3)
III		3	(2.3)	2	(66.7)	1	(33.3)	1	(33.3)	2	(66.7)	1	(33.3)	2	(66.7)
IV		15	(11.6)	7	(46.7)	8	(53.3)	3	(20.0)	12	(80.0)	9	(60.0)	6	(40.0)
**L-Category**	*n p*-Value _(a)_	145		145			0.619	145			**0.003** *	145			0.620
L0		73	(50.3)	37	(50.7)	36	(49.3)	48	(65.8)	25	(34.2)	34	(46.6)	39	(53.4)
L1		72	(49.7)	33	(45.8)	39	(54.2)	29	(40.3)	43	(59.7)	37	(51.4)	35	(48.6)
**V-Category**	*n p*-Value _(a)_	144		144			0.696	144			1.000	144			0.697
V0		110	(76.4)	54	(49.1)	56	(50.9)	59	(53.6)	51	(46.4)	53	(48.2)	57	(51.8)
V1		34	(23.6)	15	(44.1)	19	(55.9)	18	(52.9)	16	(47.1)	18	(52.9)	16	(47.1)
**Pn-Category**	*n p*-Value _(a)_	124		124			1.000	124			0.544	124			1.000
Pn0		12	(9.7)	6	(50.0)	6	(50.0)	8	(66.7)	4	(33.3)	6	(50.0)	6	(50.0)
Pn1		112	(90.3)	54	(48.2)	58	(51.8)	59	(52.7)	53	(47.3)	52	(46.4)	60	(53.6)
**Grading**	*n p*-Value _(a)_	153		154			0.054	154			0.927	154			0.075
G1		8	(5.2)	7	(87.5)	1	(12.5)	4	(50.0)	4	(50.0)	4	(50.0)	4	(50.0)
G2		78	(51.0)	34	(43.6)	44	(56.4)	40	(51.3)	38	(48.7)	30	(38.5)	48	(61.5)
G3		67	(43.8)	30	(44.8)	37	(55.2)	32	(47.8)	35	(52.2)	38	(56.7)	29	(43.3)
**R-Status**	*n p*-Value _(a)_	141		141			0.072	141			0.761	141			0.320
R0		108	(76.6)	57	(52.8)	51	(47.2)	55	(50.9)	53	(49.1)	55	(50.9)	53	(49.1)
R1		32	(22.7)	11	(34.4)	21	(65.6)	18	(56.3)	14	(43.8)	13	(40.6)	19	(59.4)
R2		1	(0.7)	0	(0.0)	1	(100.0)	1	(100.0)	0	(0.0)	0	(0.0)	1	(100.0)
**Diabetes status**	*n p*-Wert _(a)_	101		101			0.874	101			0.936	101			0.326
no diabetes		73	(72.3)	33	(45.2)	40	(54.8)	38	(52.1)	35	(47.9)	33	(45.2)	40	(54.8)
type 2 diabetes		23	(22.8)	9	(39.1)	14	(30.9)	13	(56.5)	10	(43.5)	7	(30.4)	16	(69.6)
postoperative diabetes		5	(5.0)	2	(40.0)	3	(60.0)	3	(60.0)	2	(40.0)	3	(60.0)	2	(40.0)
**Overall Survival [Months]**	*p*-Value _(c)_			155			0.965	155			0.494	155			0.378
Total/Events/Censored		155/107/48		75/51/24		80/56/24		76/50/26		79 /57/22		72/52/20		83/55/28	
Median Survival		14.9 ± 1.9		15.0 ± 1.3		13.7 ± 3.9		12.9 ± 1.8		19.1 ± 4.1		13.5 ± 2.3		14.9 ± 4.1	
95% C.I.		11.1–18.6		12.4–17.5		6.0–21.4		9.4–16.4		11.1–27.2		9.0–17.9		6.8–23.0	
**Tumor Specific Survival [Months]**	*p*-Value _(c)_			153			0.804	153			0.512	153			0.478
Total/Events/Censored		153/100/53		73/48/25		80/52/28		75/47/28		78/53/25		70/47/23		83/53/30	
Median Survival		15.7 ± 1.9		15.7 ± 1.7		15.0 ± 4.3		13.7 ± 2.0		19.2 ± 5.0		15.1 ± 1.7		18.7 ± 4.8	
95% C.I.		11.9–19.4		12.3–19.1		6.6–23.4		9.8–17.5		9.4–28.9		11.7–18.3		9.3–28.0	

_(a)_ Fisher’s exact test, _(b)_ Kendall’s tau test, _(c)_ Log-rank test. * *p* values having lost significance according to the Siemes (Benjamini-Hochberg) procedure for multiple testing.

**Table 2:** Correlation between clinicopathological patient characteristics and the expression of the insulin-like growth factor receptor 1 (IGF1R) in cancer cells in PDAC tissues.

		Total		Cytoplasmic IGF1R Expression				Membranous IGF1R Expression			
				Low HScore < 10		High HScore ≥ 10		Low HScore < 12		High HScore ≥ 12	
		*n*	(%)	*n*	(%)	*n*	(%)	*n*	(%)	*n*	(%)
**Gender**	*n p*-Value _(a)_	160		160			0.874	160			0.752
Male		80	(50.0)	39	(48.8)	41	(51.2)	38	(47.5)	42	(52.5)
Female		80	(50.0)	37	(46.3)	43	(53.8)	41	(51.2)	39	(48.8)
**Age Group**	*n p*-Value _(a)_	160		160			1.000	160			1.000
<68.3 years		80	(50.0)	38	(47.5)	42	(52.5)	39	(48.8)	41	(51.2)
≥68.3 years		80	(50.0)	38	(47.5)	42	(52.5)	40	(50.0)	40	(50.0)
**T-Category**	*n p*-Value _(b)_	147		147			0.959	147			0.202
T1		4	(2.7)	3	(75.0)	1	(25.0)	2	(50.0)	2	(50.0)
T2		9	(6.1)	2	(22.2)	7	(77.8)	1	(11.1)	8	(88.9)
T3		130	(88.4)	65	(50.0)	65	(50.0)	70	(53.8)	60	(46.2)
T4a/b		4	(2.7)	1	(25.0)	3	(75.0)	1	(25.0)	3	(75.0)
**lymph node ratio**	*n p*-Value _(a)_	147		147			0.622	147			1.000
low (<0.133)		73	(49.7)	37	(50.7)	36	(49.3)	37	(50.7)	36	(49.3)
high (≥0.133)		74	(50.3)	34	(45.9)	40	(54.1)	37	(50.0)	37	(50.0)
***N*-Category**	*n p*-Value _(a)_	147		147			0.832	147			0.395
N0		27	(18.4)	14	(51.9)	13	(48.1)	16	(59.3)	11	(40.7)
N+ (N1, N2)		120	(81.6)	57	(47.5)	63	(52.5)	58	(48.3)	62	(51.7)
**M-Category**	*n p*-Value _(a)_	135		135			0.590	135			0.419
M0		120	(88.9)	59	(49.2)	61	(50.8)	63	(52.5)	57	(47.5)
M1		15	(11.1)	6	(40.0)	9	(60.0)	6	(40.0)	9	(60.0)
**UICC Stage**	*n p*-Value _(b)_	129		129			0.647	129			0.175
IA		3	(2.3)	2	(66.7)	1	(33.3)	2	(66.7)	1	(33.3)
IB		2	(1.6)	0	(0.0)	2	(100.0)	0	(0.0)	2	(100.0)
IIA		14	(10.9)	7	(50.0)	7	(50.0)	10	(71.4)	4	(28.6)
IIB		92	(71.3)	46	(50.0)	46	(50.0)	47	(51.1)	45	(48.9)
III		3	(2.3)	1	(33.3)	2	(66.7)	1	(33.3)	2	(66.7)
IV		15	(11.6)	6	(40.0)	9	(60.0)	6	(40.0)	9	(60.0)
**L-Category**	*n p*-Value _(a)_	145		145			0.068	145			0.741
L0		73	(50.3)	41	(56.2)	32	(43.8)	38	(52.1)	35	(47.9)
L1		72	(49.7)	29	(40.3)	43	(59.7)	35	(48.6)	37	(51.4)
**V-Category**	*n p*-Value _(a)_	144		144			0.434	144			0.845
V0		110	(76.4)	55	(50.0)	55	(50.0)	56	(50.9)	54	(49.1)
V1		34	(23.6)	14	(41.2)	20	(58.8)	16	(47.1)	18	(52.9)
**Pn-Category**	*n p*-Value _(a)_	124		124			1.000	124			1.000
Pn0		12	(9.7)	7	(58.3)	5	(41.7)	7	(58.3)	5	(41.7)
Pn1		112	(90.3)	60	(53.6)	52	(46.4)	60	(53.6)	52	(46.4)
**Grading**	*n p*-Value _(a)_	153		154			0.277	154			0.327
G1		8	(5.2)	6	(75.0)	2	(25.0)	6	(75.0)	2	(25.0)
G2		78	(51.0)	35	(45.6)	43	(54.4)	39	(50.0)	39	(50.0)
G3		67	(43.8)	32	(47.8)	35	(52.2)	31	(46.3)	36	(53.7)
**R-Status**	*n p*-Value _(a)_	141		141			0.160	141			0.547
R0		108	(76.6)	48	(44.4)	60	(55.6)	53	(49.1)	55	(50.9)
R1		32	(22.7)	19	(59.4)	13	(40.6)	18	(56.3)	14	(43.8)
R2		1	(0.7)	0	(0.0)	1	(100.0)	0	(0.0)	1	(100.0)
**Diabetes status**	*n p*-Wert _(a)_	101		101			0.277	101			0.622
no diabetes		73	(72.3)	34	(46.6)	39	(53.4)	33	(45.2)	40	(54.8)
type 2 diabetes		23	(22.8)	15	(65.2)	8	(34.8)	13	(56.5)	10	(43.5)
postoperative diabetes		5	(5.0)	2	(40.0)	3	(60.0)	3	(60.0)	2	(40.0)
**Overall Survival [Months]**	*p*-Value _(c)_			155			0.671	155			0.194
Total/Events/Censored		155/107/48		74/52/22		81/55/26		75/51/24		80/56/24	
Median Survival		14.9 ± 1.9		15.7 ± 2.1		14.7 ± 3.5		15.7 ± 2.6		13.5 ± 2.0	
95% C.I.		11.1–18.6		11.7–19.7		7.9–21.4		10.5–20.8		9.6–17.4	
**Tumor Specific Survival [Months]**	*p*-Value _(c)_			153			0.832	153			0.367
Total/Events/Censored		153/100/53		73/49/24		80/51/29		74/49/25		79/51/28	
Median Survival		15.7 ± 1.9		15.7 ± 2.0		15.0 ± 3.0		15.7 ± 2.6		14.9 ± 2.8	
95% C.I.		11.9–19.4		11.8–19.6		9.0–21.0		10.6–20.7		9.4–20.4	

_(a)_ Fisher’s exact test, _(b)_ Kendall’s tau test, _(c)_ Log-rank test.

**Table 3:** Correlation between the expression of the insulin-like growth factor receptor 1 (IGF1R) and the insulin receptor (IR) in cancer cells and vasculature.

	Tumoral Cytoplasmic IGF1R Expression			Tumoral Membranous IGF1R Expression		
	Low (HScore < 10)	High (HScore ≥ 10)	*p*-Value (a)	Low (HScore < 12)	High (HScore ≥ 12)	*p*-Value (a)
	*n* (%)	*n* (%)		*n* (%)	*n* (%)	
**Vascular IR expression**						
**low (HScore <135)**	45 (58.4)	32 (41.6)	0.011 *	46 (59.7)	31 (40.3)	**0.017** *
**high (HScore ≥ 135)**	31 (37.3)	52 (62.7)		33 (39.8)	50 (60.2)	
**Cytoplasmic IR expression**						
**low (HScore < 101)**	40 (50.6)	39 (49.4)	0.527	40 (50.6)	39 (49.4)	0.874
**high (HScore ≥ 101)**	36 (44.4)	45 (55.6)		39 (48.1)	42 (51.9)	
**Membranous IR expression**						
**low (HScore < 120)**	33 (44.0)	42 (56.0)	0.431	37 (49.3)	38 (50.7)	1000
**high (HScore ≥ 120)**	43 (50.6)	42 (49.4)		42 (49.4)	43 (50.6)	

(a) Fisher’s exact. * *p* values having lost significance according to the Siemes (Benjamini-Hochberg) procedure for multiple testing.

**Table 4:** Comparison of insulin receptor (IR) and IGF1 receptor expression between pancreatic cancer samples and corresponding intratumoral PanIN lesions.

	**PanIN Low Grade** **Cytoplasmic IR Expression**			**PanIN High Grade** **Cytoplasmic IR Expression**		
	**Low** **(HScore < 101)**	**High** **(HScore ≥ 101)**	***p*** **-Value (a)**	**Low** **(HScore < 101)**	**High** **(HScore ≥ 101)**	***p*** **-Value (a)**
**Tumoral cytoplasmic IR expression**	*n* (%)	*n* (%)		*n* (%)	*n* (%)	
**low (HScore < 101)**	7 (43.8)	9 (56.3)	0.716	1 (20.0)	4 (80.0)	0.301
**high (HScore ≥ 101)**	5 (33.3)	10 (66.7)		5 (55.6)	4 (44.4)	
	**PanIN Low Grade** **Membranous IR Expression**			**PanIN High Grade** **Membranous IR Expression**		
	**Low** **(HScore < 120)**	**High** **(HScore ≥ 120)**	***p*** **-Value (a)**	**Low** **(HScore < 120)**	**High** **(HScore ≥ 120)**	***p*** **-Value (a)**
**Tumoral membranous IR expression**	*n* (%)	*n* (%)		*n* (%)	*n* (%)	
**low (HScore < 120)**	7 (53.8)	6 (46.2)	0.481	3 (100)	0 (0.0)	0.258
**high (HScore ≥ 120)**	7 (38.9)	11 (61.1)		6 (54.5)	5 (45.5)	
	**PanIN Low Grade** **Cytoplasmic IGF1R Expression**			**PanIN High Grade** **Cytoplasmic IGF1R Expression**		
	**Low** **(HScore < 10)**	**High** **(HScore ≥ 10)**	***p*** **-Value (a)**	**Low** **(HScore < 10)**	**High** **(HScore ≥ 10)**	***p*** **-Value (a)**
**Tumoral cytoplasmic IGF1R expression**	*n* (%)	*n* (%)		*n* (%)	*n* (%)	
**low (HScore < 10)**	13 (61.9)	8 (38.1)	0.441	6 (85.7)	1 (14.3)	0.103
**high (HScore ≥ 10)**	4 (40.0)	6 (60.0)		2 (28.6)	5 (71.4)	
	**PanIN Low Grade** **Membranous IGF1R Expression**			**PanIN High Grade** **Membranous IGF1R Expression**		
	**Low** **(HScore <12)**	**High** **(HScore ≥ 12)**	***p*** **-Value (a)**	**Low** **(HScore <12)**	**High** **(HScore ≥ 12)**	***p*** **-Value (a)**
**Tumoral membranous IGF1R expression**	*n* (%)	*n* (%)		*n* (%)	*n* (%)	
**low (HScore <12)**	15 (75.0)	5 (25.0)	0.423	6 (100)	0 (0.0)	**0.031** *
**high (HScore ≥ 12)**	6 (54.5)	5 (45.5)		3 (37.5)	5 (62.5)	

(a) Fisher’s exact. * *p* values having lost significance according to the Siemes (Benjamini-Hochberg) procedure for multiple testing.

## Data Availability

The original data supporting the reported results can be provided upon request.
